# Intracranial lateralization bias observed in the presence of symmetrical hearing thresholds

**DOI:** 10.1121/10.0006720

**Published:** 2021-10-15

**Authors:** Matthew J. Goupell, Virginia Best, H. Steven Colburn

**Affiliations:** 1 Department of Biomedical Engineering, Boston University, Boston, Massachusetts 02215, USA; 2 Department of Speech, Language, and Hearing Sciences, Boston University, Boston, Massachusetts 02215, USA goupell@umd.edu, ginbest@bu.edu, colburn@bu.edu

## Abstract

It is generally assumed that listeners with normal audiograms have relatively symmetric hearing, and more specifically that diotic stimuli (having zero interaural differences) are heard as centered in the head. While measuring intracranial lateralization with a visual pointing task for tones and 50-Hz-wide narrowband noises from 300 to 700 Hz, examples of systematic and large (>50% from midline to the ear) lateralization biases were found. In a group of ten listeners, five showed consistent lateralization bias to the right or left side at all or a subset of frequencies. Asymmetries in hearing, not apparent in audiometric thresholds, may explain these lateralization biases.

## 1. Introduction

When a sound source is directly in front of or behind a listener, and thus is equidistant to the two ears, the sound reaching the ears will have zero interaural time difference (ITD) and interaural level difference (ILD). It is generally assumed that the combination of zero ITD and ILD is mapped to the center of the acoustic reference system and is perceived as “centered” by the listener. This idea is broadly supported by dichotic listening experiments, in which stimuli are presented over headphones such that ITD and ILD can be manipulated systematically. In such experiments, sounds are typically heard inside the head rather than in external space (Best *et al.*, 2020), with stimuli having zero ITD and ILD being perceived at or near the center of the head, and non-zero ITDs and/or ILDs causing a shift in intracranial position to one side. If a diotic sound is not perceived as centered, a common interpretation is that the listener has some asymmetry in their hearing thresholds (Koehnke *et al.*, 1995; Fitzgerald *et al.*, 2015) or that an asymmetry has occurred because of the equipment [e.g., from the headphone placement; Hershkowitz and Durlach (1969)]. Contrary to these interpretations, here we report that an intracranial lateralization bias can occur for listeners with symmetrical thresholds in the range of normal hearing, and without evidence that it is caused by the experimental equipment.

## 2. Methods

Four highly experienced psychoacousticians (S1–S4, including two of the authors S3 and S4; 29–41 years) and six naive and young (18–23 years) listeners participated in this study. All listeners had audiometrically normal hearing, defined as <20 dB HL at octave frequencies from 0.25 to 8 kHz with interaural asymmetries ≤10 dB at any frequency (Table 1). Audiometric thresholds were collected for each ear independently using a Hughson-Westlake procedure, which was done by a researcher in a double-walled soundproof booth using a calibrated audiometer (Grason-Stadler AudioStarPro; Eden Prairie, MN).

**Table 1.** Audiometric thresholds for the right and left ears, and their difference. The right-left difference in threshold at 500 Hz is highlighted in bold.

		Frequency (Hz)					
Listener		250	500	1000	2000	4000	8000
S1	Right	5	5	5	5	0	15
	Left	5	0	0	−5	−5	15
	Difference	0	**5**	5	10	5	0
S2	Right	−10	0	5	0	0	5
	Left	−5	0	−5	0	5	−5
	Difference	−5	**0**	10	0	−5	10
S3	Right	−5	−5	0	−10	−5	10
	Left	−5	−5	0	−5	−5	5
	Difference	0	**0**	0	−5	0	5
S4	Right	5	5	0	0	0	10
	Left	0	5	0	0	0	15
	Difference	5	**0**	0	0	0	−5
S5	Right	0	−5	0	−5	−5	5
	Left	5	0	−5	0	0	5
	Difference	−5	**−5**	5	−5	−5	0
S6	Right	5	5	5	5	−5	15
	Left	10	5	5	5	0	15
	Difference	−5	**0**	0	0	−5	0
S7	Right	5	5	5	5	5	10
	Left	10	5	5	0	0	0
	Difference	−5	**0**	0	5	5	10
S8	Right	10	5	0	5	−5	0
	Left	5	10	0	5	0	5
	Difference	5	**−5**	0	0	−5	−5
S9	Right	5	5	0	5	−5	5
	Left	10	10	5	5	0	10
	Difference	−5	**−5**	−5	0	−5	−5
S10	Right	5	10	0	0	−5	−10
	Left	5	5	−5	−5	−10	−10
	Difference	0	**5**	5	5	5	0

The experiment was controlled using matlab (MathWorks, Natick, MA) software on a personal computer. The stimuli were generated in matlab, and delivered from a real-time processor and headphone buffer (RP2.1, HB7; Tucker-Davis Technologies, Alachua, FL) over a pair of circumaural headphones (HD 265 Sennheiser, Hannover, Germany). Listeners sat in a double-walled sound attenuated chamber (IAC, North Aurora, IL). A graphical user interface implemented in matlab was displayed on a computer monitor and was used to collect listener responses.

The stimuli were pure tones and 50-Hz-wide narrowband noises. The tones and noises had center frequencies (CFs) of 300, 400, 500, 600, or 700 Hz. The narrowband noises were generated in the frequency domain and thus initially had infinite steepness. Each tone or narrowband noise was 600 ms in duration and was temporally shaped by a Tukey window with a rise-fall time of 250 ms. The temporal shaping was applied diotically after the introduction of the ITD so that there would be a diminished effect of the onset ITD. The phases of the sinusoids were zero. The level of each narrowband noise or tone was 63 dB SPL. The ITD was set to 0, ±125, ±250, ±375, ±500, ±625, ±750, or ±1500 *μ*s. Positive ITDs were defined as right leading. For each CF, however, the ITD range that was ultimately analyzed was limited to exclude values with an interaural phase difference beyond ±7*π*/8 to avoid ambiguities occurring at the *π* boundary. ILDs were always 0 dB.

Listener responses were collected for an intracranial lateralization task using a visual reference scale (Sayers, 1964; Yost, 1981; Zhang and Hartmann, 2006). Lateral position was indicated by clicking on bars that were visualized across a face presented on the screen; the position of each response on a bar was converted to a continuous scale that places –100% at the left ear, 0% at the center, and +100% at the right ear. Therefore, the scale represents the percentage of linear distance from the midline to the ear. Listeners could indicate up to three image locations across three bars (Fitzgerald *et al.*, 2015). Listeners could repeat stimuli as needed. Neither training nor feedback were provided.

The 80 different stimuli (2 stimulus types × 5 CFs × 8 ITDs) were randomized within a single block of trials. The block contained two trials per condition (one left-leading and one right-leading for non-zero ITDs) for a total of 160 trials. At least ten blocks were completed. After each block, the headphones were taken off and reversed, which was visually confirmed by an experimenter, and the designation of left/right in the stimuli was reversed accordingly. The purpose of this was to remove any transducer-based bias and to minimize effects of headphone position [see also Moushegian and Jeffress (1959) and Harris (1960)].

Data were averaged for each value of ITD; for trials in which more than one response location was indicated, they were treated as separate observations and both locations were included in the average. However, more than one response was infrequent and thus likely had negligible impact on the results, representing 3.3% of the trials for the conditions within ±7π/8 interaural phase difference limit. A four-parameter cumulative Gaussian function was fit to the data points for each condition and listener; the fit had the form: $y=A\left[1+\text{erf}\left[\left(x-\mu_{x}\right)/\sqrt{2}\sigma \right]\right]-\mu_{y},$ where *x* is the value of ITD and *A*, *σ*, *μ_X_*, and *μ_Y_* are the four free parameters that were optimized to fit the data (Fitzgerald *et al.*, 2015).

## 3. Results

**Fig. 1.**
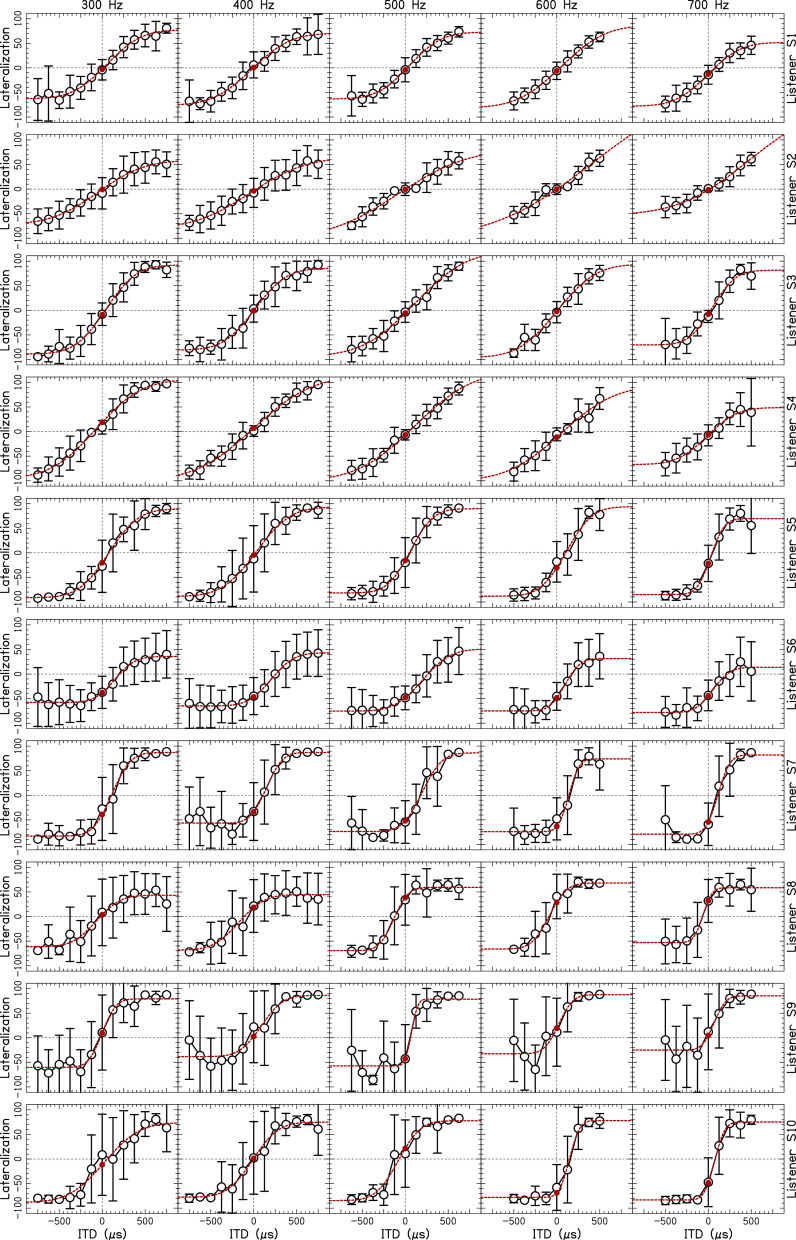
Individual lateralization responses in percentage from midline to left (negative) or right (positive) ear as a function of ITD for tones. Error bars represent ±1 standard deviation across the trials. Dashed lines represent cumulative Gaussian fits to the data. The filled solid circle represents the y-intercept (i.e., the lateralization bias) at 0-*μ*s ITD.

Figure 1 shows the data for pure tones, with one panel per CF and individual listener. The functional fits to the data points had a median adjusted R^2^ value of 0.99. The worst adjusted R^2^ value was 0.85 for listener S9 for the 600-Hz tone.

Listeners S1–S4 (the highly experienced listeners) showed well behaved data: lateralization curves that transition from left to right with increasing ITD (as indicated by the dashed lines in Fig. 1), small lateralization biases at zero ITD (as indicated by the filled circles on the dashed lines), and relatively good consistency in their lateralization responses (as indicated by the small error bars, representing the standard deviation of the measurements). Listener S5 also showed well-behaved lateralization curves, but there was a systematic lateralization bias to the left side at zero ITD for all frequencies. This lateralization bias was relatively small considering the size of the error bars for this listener. Listener S6 showed a similar pattern to S5, although with a more extreme left-side bias such that the lateralization bias was larger than the error bars for this listener. Listener S7 showed the largest lateralization bias, with an average lateralization value of –59% at zero ITD for the 700-Hz tone. Listener S8 gave centered responses for zero ITD at 300 Hz, but showed an increasing rightward lateralization bias as frequency increased to 700 Hz. Listener S9 had some of the most variable responses in the experiment and some evidence of a leftward lateralization bias at 500 Hz. Listener S10 was also variable, but lateralization curves were relatively systematic as a function of ITD. This listener had a lateralization bias to the left side at 600 and 700 Hz. Finally, the lateralization biases were unrelated to the difference in audiometric thresholds at measured at 500 Hz (Table 1).

**Fig. 2.**
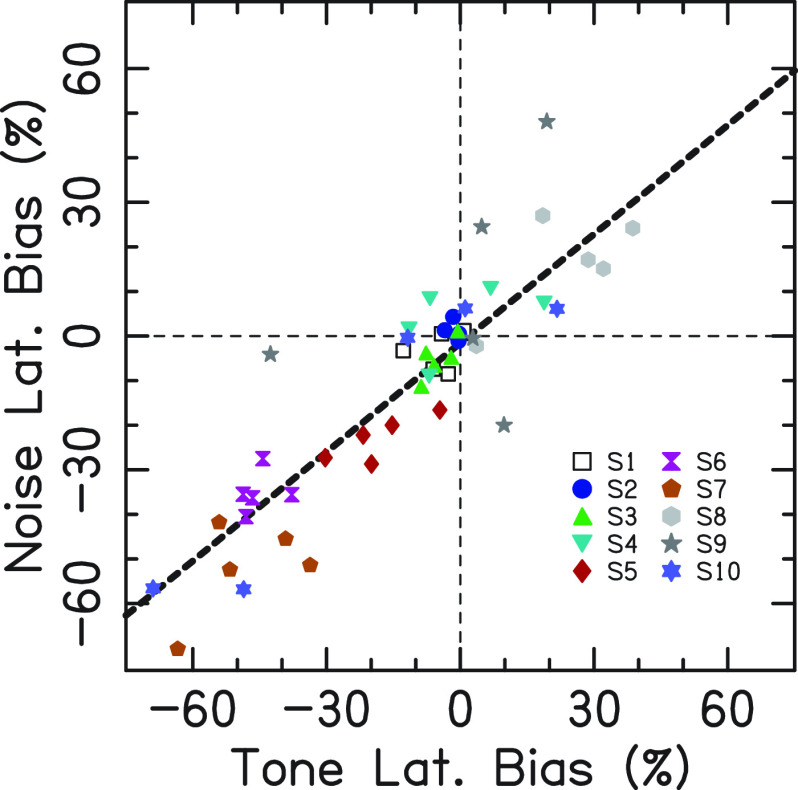
Lateralization bias in percentage from midline to left (negative) or right (positive) ear for tones (*x* axis) and narrowband noises (*y* axis). There are five points per listener (for the five CFs), and each listener is represented by a different symbol.

Figure 2 shows the lateralization bias at zero ITD for tones (i.e., the filled circles in Fig. 1) and narrowband noises. The biases were clearly related across the two stimulus types, usually observed with the same sign in the same listeners. There are notable differences across listeners with respect to the sign and magnitude of the biases. S1–S4 demonstrated near zero bias. Listeners S5, S6, and S7 demonstrated negative (leftward) lateralization biases. Listener S8 demonstrated positive (rightward) lateralization biases at four frequencies, but not at 300 Hz (see Fig. 1). Listener S9 also demonstrated mostly positive lateralization biases; however, they were the most inconsistent across stimulus type and frequency. Listener S10 demonstrated a negative lateralization bias for only two frequencies, 600 and 700 Hz.

## 4. Discussion

This experiment showed that stimuli delivered with zero ITD and ILD were not always perceived as centered in the head for all listeners. Rather, some listeners demonstrated a systematic intracranial lateralization bias (Figs. 1 and 2). Importantly, our listeners all had audiometrically normal hearing thresholds and interaural asymmetries of ≤5 dB in the frequency range of interest. Some listeners were biased across all frequencies (S5, S6, and S7). When averaged across both stimuli and all CFs, the most extreme biases were observed for listener S6 (average lateralization bias of −40.1%) and listener S7 (average lateralization bias of −50.3%). Using the x-intercept value of the fits, these two listeners perceived centered images for stimuli with an ITD of 223 and 130 *μ*s, respectively [approximately 25° and 12.5° of azimuthal angle according a spherical head model; Woodworth and Schlosberg (1954)]. Two listeners demonstrated a bias at a subset of frequencies (S8 and S10). If the biases occurred by chance, it would be highly unlikely that they should occur at only some frequencies. Instead, these biases were repeatable using different narrowband stimuli (tones vs narrowband noises; Fig. 2). Only one listener appeared to have such variable data within and across conditions that their reported lateralization biases could have been a result of inconsistency in performing the task (S9, Fig. 2). Finally, fits to the data points provide a clear indication that some of the listeners experienced systematic lateralization biases that affected stimuli with non-zero ITDs as well as the “midline” zero ITD stimulus (dashed lines in Fig. 1).

When we first observed these intracranial lateralization biases in preliminary experiments, our initial assumption was that they were artifacts of the experimental setup rather than true effects inherent to the listeners. Thus, in the final experiment, we took several steps to minimize any potential contributions of the experimental setup. To dismiss effects related to asymmetries in the transducers, we adopted the procedure of reversing the headphones after each block (Moushegian and Jeffress, 1959; Harris, 1960). To dismiss effects related to the testing environment, we confirmed that there were no sources of noise within the sound booth that could have biased responses. We also ensured that the testing station was at a location with no asymmetries in the visual field of view (e.g., the location of windows and the door). Given that systematic biases persisted despite these precautions, we believe that they must stem from other procedural or listener-related factors.

While we are not aware of any other *formal* reports of this phenomenon, there appears to be evidence of intracranial lateralization bias in the literature. Historically, intracranial lateralization biases have been treated as an unwanted source of variability and a variety of approaches have been taken to minimize their influence. For example, several investigators identified headphone placement as a source of variability and would reset the transducers multiple times until a centered image was achieved for a diotic stimulus before commencing experimental trials [e.g., Hershkowitz and Durlach (1969) and Domnitz and Colburn (1977)]. In cases where center could not be achieved, deviations are reported to be small [e.g., within 5% of the scale; Yost (1981)]. Other studies used an intracranial pointing task with a “calibrated” acoustic pointer that was lateralized via ITD or ILD. In this procedure, small lateralization biases are often corrected for in the calibration procedure by applying an appropriate binaural offset to every stimulus. For example, acoustic pointers, where the intracranial location of a matching stimulus is manipulated using an ILD, have been used in a long series of ITD lateralization studies [e.g., Bernstein and Trahiotis (1985), (2003), (2012)]. The authors reported the ILD offset needed for centering to be generally small, up to a few dB, but sometimes larger [Bernstein and Trahiotis (1985); see footnote 6]. Such a calibration approach by definition obscures any lateralization biases in the published data.

The visibility of intracranial lateralization biases in the literature may also have been affected by listener characteristics, recruitment, and retention. Many classic lateralization studies used a very small number of listeners that often included “expert” listeners [e.g., Saberi (1998)]. It was also not uncommon for listeners to be excluded from an experiment if their data were difficult to interpret (McFadden *et al.*, 1973). In the current study, it was notable that the listeners with most experience with psychoacoustical experiments (S1–S4) provided the most reliable and unbiased data. Two of listeners were authors, and were aware of the distribution of the stimuli, even though they could not know the details of any individual stimulus given the randomization of the conditions. It is possible that knowledge about the stimulus distribution reduced the chances of observing a lateralization bias in these listeners. More generally, expert listeners may be better at mapping sounds via probabilities of stimuli and range provided to them (Poulton, 1979) than less experienced listeners.

Several of our own recent studies have utilized the same lateralization task using a visual reference system and inexperienced listeners. While we reported no average lateralization biases, we did observe biases in individual normal-hearing listeners that were as large as 4 dB of ILD (Stakhovskaya and Goupell, 2017; Anderson *et al.*, 2019). A majority of previous intracranial lateralization studies do not include individual lateralization curves, only those averaged over listeners and/or conditions, making it difficult to determine the true prevalence of lateralization biases at specific frequencies.

The question remains as to why these biases occur in listeners with audiometrically normal hearing. One possibility is that these listeners have small asymmetries in hearing thresholds, not easily observable given the resolution of an audiogram. Another possibility is that there may be asymmetries in suprathreshold hearing that lead to across ear differences (in loudness, quality, etc.) and an imbalanced perception of diotic suprathreshold stimuli. One could even speculate that the biases represent asymmetric presentations of “hidden” hearing loss (Budak *et al.*, 2021).

To further understand the lateralization bias phenomenon and its significance, we believe there are several important next steps. First, to better understand the prevalence of lateralization biases, larger numbers of naive listeners are needed, and the reporting of data for individual listeners and conditions is necessary. Second, follow-up experiments should include a more extensive audiological workup beyond threshold testing to determine if there are any audiological indicators that may predict lateralization biases. If testing occurs over multiple days, thresholds should be verified at each visit, not just the initial visit. Third, it would be informative to compare biases across a range of stimulus types and tasks, including ILD headphone lateralization, left-right discrimination, and free-field sound localization tasks. Fourth, it would be useful to determine the extent to which the observability of lateralization biases depend on the available range of responses and the listener's experience with the task. Fifth, it would be important to determine if lateralization biases have broader functional consequences in situations where good spatial hearing is important (e.g., free-field sound localization of broadband stimuli like speech, spatial release from masking).
